# Combinatorial Pattern Discovery Approach for the Folding Trajectory Analysis of a *β*-Hairpin

**DOI:** 10.1371/journal.pcbi.0010008

**Published:** 2005-06-24

**Authors:** Laxmi Parida, Ruhong Zhou

**Affiliations:** 1 Computational Biology Center, IBM Thomas J. Watson Research Center, Yorktown Heights, New York, United States of America; 2 Department of Chemistry, Columbia University, New York, New York, United States of America; Peking University, China

## Abstract

The study of protein folding mechanisms continues to be one of the most challenging problems in computational biology. Currently, the protein folding mechanism is often characterized by calculating the free energy landscape versus various reaction coordinates, such as the fraction of native contacts, the radius of gyration, RMSD from the native structure, and so on. In this paper, we present a combinatorial pattern discovery approach toward understanding the global state changes during the folding process. This is a first step toward an unsupervised (and perhaps eventually automated) approach toward identification of global states. The approach is based on computing biclusters (or patterned clusters)—each cluster is a combination of various reaction coordinates, and its signature pattern facilitates the computation of the Z-score for the cluster. For this discovery process, we present an algorithm of time complexity *c∈R*O**((*N + nm*) log *n*), where *N* is the size of the output patterns and (*n* ×* m*) is the size of the input with *n* time frames and *m* reaction coordinates. To date, this is the best time complexity for this problem. We next apply this to a *β*-hairpin folding trajectory and demonstrate that this approach extracts crucial information about protein folding intermediate states and mechanism. We make three observations about the approach: (1) The method recovers states previously obtained by visually analyzing free energy surfaces. (2) It also succeeds in extracting meaningful patterns and structures that had been overlooked in previous works, which provides a better understanding of the folding mechanism of the *β*-hairpin. These new patterns also interconnect various states in existing free energy surfaces versus different reaction coordinates. (3) The approach does not require calculating the free energy values, yet it offers an analysis comparable to, and sometimes better than, the methods that use free energy landscapes, thus validating the choice of reaction coordinates. (An abstract version of this work was presented at the 2005 Asia Pacific Bioinformatics Conference [1].)

## Synopsis

The study of protein folding mechanisms continues to be one of the most challenging problems in computational biology. Currently, the protein folding mechanism is often characterized by calculating the free energy landscape versus various reaction coordinates, such as the fraction of native contacts, the radius of gyration, RMSD from the native structure, and so on. In this paper, the authors present a combinatorial pattern discovery approach toward understanding the global state changes during the folding process. This is a first step toward an unsupervised (and perhaps eventually automated) approach toward identification of global states. The authors apply this approach to a *β*-hairpin folding trajectory and demonstrate that this approach extracts crucial information about protein folding intermediate states and mechanism.

## Introduction

Understanding protein folding is one of the most challenging problems in molecular biology [2–7]. The interest is not only in obtaining the final fold (generally referred to as structure prediction) [8–10] but also in understanding the folding mechanism and folding kinetics involved in the actual folding process. Many native proteins fold into unique globular structures on a very short time scale. The so-called fast folders can fold into the functional structure from random coil in microseconds to milliseconds. Recent advances in experimental techniques that probe proteins at different stages during the folding process have shed light on the nature of the folding kinetics and thermodynamics [11–17]. However, due to experimental limitations, detailed protein folding pathways remain unknown. Computer simulations performed at various levels of complexity, ranging from simple lattice models to all-atom models with explicit solvent, can be used to supplement experiment and fill in some of the gaps in our knowledge about folding mechanisms.

Large-scale simulations about protein folding with realistic all-atom models still remain a great challenge [3–5,7]. Enormous effort is needed for this grand problem; one example is the recent IBM Blue Gene project, which is aimed at building a supercomputer with hundreds-of-teraflop to petaflop computing power to tackle the protein folding problem. Meanwhile, effective analyses of the trajectory data from the protein folding simulations, either by molecular dynamics or Monte Carlo, remains yet another challenge due to the large number of degrees of freedom and the huge amount of trajectory data. [18,19] Currently, the protein folding mechanism is often characterized by calculating the free energy landscape versus the so-called reaction coordinates [3,20,21]. We and others have used various reaction coordinates [3,20,21], such as the fraction of native contacts, the radius of gyration of the entire protein, the root mean square deviation (RMSD) from the native structure, the number of *β*-strand hydrogen bonds, the number of *α*-helix turns, the hydrophobic core radius of gyration, and the principal components (PC) from principal component analysis [20,22]. Searching for better reaction coordinates is still of great interest in protein folding mechanism studies. These analyses have provided important information for a better understanding of protein folding. However, it often requires a priori knowledge about the system under study, and the free energy contour maps usually result in too much information reduction due to their limit in dimensionality, which is often as low as two or three. Thus, better or complementary analysis tools are in great demand.

It is also known that the folding process of many proteins takes the amino acid coil through different intermediate states before stabilizing on the final folded state. Therefore, a first step toward understanding the folding process is to identify these states. In this paper, we propose the use of a combinatorial pattern discovery technique to analyze protein folding trajectory data from simulation experiments. A novel aspect of the current algorithm is that it incorporates arbitrary and possibly different distribution functions of the data in each dimension and guarantees complete and accurate solution to the clustering problem. The procedure involves computations of clusters of the data: each cluster has a signature pattern describing all the elements of the cluster. The simplicity of the pattern leads to easy interpretation of and thus better understanding of the underlying processes and facilitates the computation of a Z-score for the cluster. By appropriate redundancy checks, the number of clusters is made manageably small. The results of this method are threefold. Firstly, the method is validated by comparing its results with previously published results with a free energy landscape analysis. Secondly, the method succeeds in extracting meaningful new patterns and structures that had been overlooked before. These new structures provide a better understanding of the folding mechanism of a *β*-hairpin, which is used as a case study in this paper. These new patterns also interconnect various states in existing free energy contour maps versus different reaction coordinates. This success encourages us to postulate that the automatic discovery will lead to much greater understanding of the folding process. Thirdly, the method validates the choice of reaction coordinates since the pattern discovery analysis based on these reaction coordinates compares well with the previous free energy based approaches.

## Results/Discussion

### Description of Models

**Figure 1:**
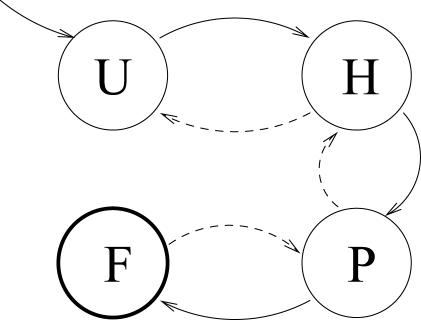
A Hypothetical State Diagram of a Folding of Protein A schema of the folding process for a small protein, a *β*-hairpin. It starts with an unfolded state, U state, undergoes to a hydrophobic core collapsed H state, and then to a partially folded P state before finally ending at the folded F state.

Well-known simulation methods exist to carry out the folding of a protein. However, it is often not sufficient to obtain a succinct understanding of the folding process. The task here is to understand the folding mechanism by recognizing structural patterns or intermediate states that the folding process goes through. For example, the folding of a small protein, a *β*-hairpin, could be understood at a global level in terms of the states shown in Figure 1. Although we would aim to understand the folding of every protein in this simplistic form, the current state of the art is far from this goal.

At each step of the simulation process, a configuration of the solvated protein can be computed. However, the simulation may be carried for nanoseconds to microseconds in units of femtoseconds (10^−15^), so the number of such intermediate configurations could easily be millions in number. Hence, the task is to identify and capture representative intermediate configurations. Since working in the structure space of the protein is extremely complex, researchers often identify a few key characteristic features of the protein, or often so-called reaction coordinates, and study the trends and variations in these reaction coordinates [21,23].

**Figure 2:**
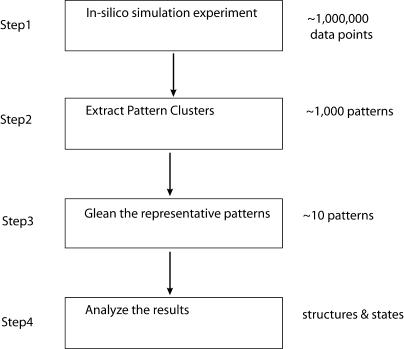
The Flowchart of the Process of Understanding a Folding Protein Step 1 starts with millions of data points obtained from the simulation experiments. Step 2 extracts the recurring patterns, reducing the size of the data to be studied down to thousands. Step 3 further reduces down this to a representative set of a handful states, which are studied in detail in Step 4. The structures are extracted, and a possible state diagram summarizing the path of the folding protein is elucidated.

In this paper, we utilize a four-step process toward understanding the folding of a protein (Figure 2). The first step involves the in silico simulation that gives rise to a large collection of data points, each point being an array of the characteristic features of the folding protein at that time point. For example, the radius of gyration or the number of hydrogen bonds could be such features. In the Results/Discussion section, we study the *β*-hairpin folding as a show case and describe seven such characteristic features that we have used previously in the study of this particular protein.

In the second step, we study these data points to extract the characteristic set of features that we call pattern clusters. Again, in the case of the *β*-hairpin, the data points are seven-dimensional, corresponding to the characteristic features of the protein at each time interval (see Table 1 for a small portion of the data as an example). In the third step, these patterns are filtered to retain the most significant ones. It is very difficult to model the significant patterns in this domain, so we have combined the second and third steps and use appropriate parameters to filter out possibly insignificant patterns: we use cluster size (in terms of rows) and the Z-scores.

**Table 1:**
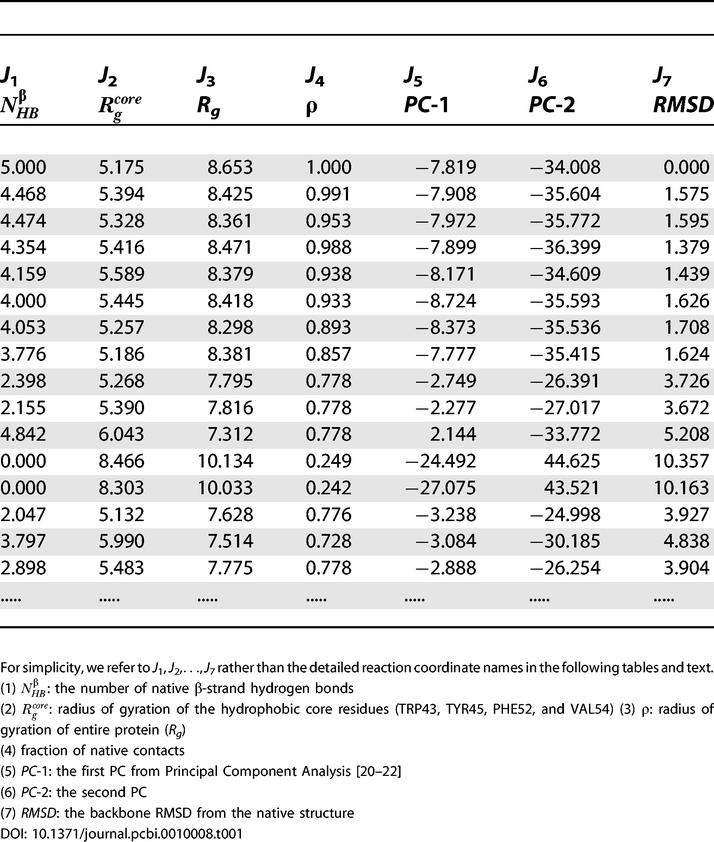
A Small Portion of the Raw Data from the REMD Sampling of the β-Hairpin Folding in Explicit Water

The fourth step is to analyze the patterns. This involves extracting the structure of the configuration using the time coordinates and studying the correlation of the different structures. For instance, one could observe that the hydrophobic core is formed before the *β*-strand hydrogen bonds, or vice versa; and one can interconnect various free energy states in different free energy surfaces by monitoring the high-dimensional (multi-column) patterns. These findings can provide a better understanding of the protein folding mechanism. Further, the time correlation between various patterns or states could be studied. For example, it is extremely useful to know which pattern or state precedes the other and by how much time.

Here, we describe in detail the second and third steps in our approach, as shown in Figure 2. We model the extraction problem as a combinatorial detection problem for at least three specific reasons: (1) The data are obtained from a replica exchange molecular dynamics (REMD) method [24] (more details below). This method is essentially a Monte Carlo method; thus, the time series is not strictly real time due to the random Monte Carlo exchange process. Also, our interest is in finding pattern clusters that are not necessarily correlated in time. (2) This emphasizes that any probabilistic (or non-deterministic) component can be isolated from the algorithm and the problem. Any high-frequency noise can be largely resolved through an introduction of a *δ* function (see below). (3) The signature pattern of the cluster helps interpret the clusters quite easily. Also, in comparison to the straightforward grouping or clustering algorithms in previous publications [21,25], this provides a complete and efficient (in linear time) method to find the signature patterns. It must be pointed out that this is the critical reason why we chose to use this method, since this enables us to have a tighter control on an acceptable cluster that is also meaningful in terms of the folding process.

A small but important protein system has been selected as an example to demonstrate our approach to understanding the folding process. This small protein is a 16-residue *β*-hairpin (GEWTYDDATKTFTVTE) from the C-terminus of protein G (residues 41–56 of PDB file 2gb1.pdb). Its folding mechanism and folding free energy states have been studied extensively in previous works [21,23]. The current study will use our new approach to analyzing the existing trajectories from the previous REMD simulations in explicit solvent [21,24]. The REMD method couples molecular dynamics trajectories with a temperature-exchange Monte Carlo process for efficient sampling of the conformational space. In this method, replicas are run in parallel at a sequence of temperatures ranging from the desired temperature to a high temperature at which the replica can easily surmount the energy barriers. From time to time, the configurations of neighboring replicas are exchanged and this exchange is accepted by a Metropolis acceptance criterion that guarantees the detailed balance. Because the high-temperature replica can traverse high-energy barriers, this provides a mechanism for the low-temperature replicas to overcome the quasi-ergodicity they would otherwise encounter in a single-temperature replica.

This *β*-hairpin has received much attention recently from both experimental and theoretical fronts [11,13,14,18,20,26–30]. The *β*-sheets and *α*-helices are the key secondary structures in proteins. It is believed that understanding the folding of these elements will be a foundation for investigating larger and more complex structures. The study of isolated *β-*sheets has for a long time been limited by the lack of an amenable experimental system. The breakthrough experiments by Serrano [11] and Eaton [13] groups have recently established this *β*-hairpin as the system of choice to study *β-*sheets in isolation. These pioneering experiments inspired a number of theoretical works on this system with various models [18,20,21,26,27,31,32]. However, there are still a number of important aspects that remain controversial, such as the relative importance and time-sequential order between the *β*-strand hydrogen bonds formation and the hydrophobic core formation, and the existence of *α*-helical intermediates during the folding.

### Simulation Parameters

In this study, an all-atom model—The Optimized Potential for Liquid Simulations-All-Atom force field [33] with an explicit solvent model, Simple Point Charge model [34]—is used for the description of the protein solvated in water. A total of 64 replicas of the solvated system consisting of 4,342 atoms is simulated with temperatures spanning from 270 K to 695 K. For each replica, a 3-nanosecond molecular dynamic simulation is run with replica exchanges attempted every 400 femtoseconds. The reader is directed to [21,23] for details of this simulation. For each conformation, seven different reaction coordinates are used (Table 1). There are a total of about 20,000 conformations saved for each replica. Table 1 lists a small portion of the data for the replica at 310 K, which is the biological temperature.

These simulations have revealed a hydrophobic-core-driven folding mechanism from free energy contour map analysis [21]. Since this is a well-studied system and a large amount of data is available, comparisons with other analysis tools, such as the free energy contour map analysis, might be easier and more straightforward. Various reaction coordinates obtained from previous runs serve as the starting point.

### Discovery Parameters

Although we developed the framework for a very general *δ* function, for simplicity, in this section we treat *δ*(*x*) to be a constant function. Thus, *δ*(*x*) = *c* for some constant 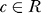 for each *x*. The *δ* functions for each column of Table 1 is given as follows: *δ*_1_(*x*) = 0.2, *δ*_2_(*x*) = 0.6, *δ*_3_(*x*) = 0.35, *δ*_4_(*x*) = 0.15, *δ*_5_(*x*) = 5.0, *δ*_6_(*x*) = 16.5, *δ*_7_(*x*) = 1.0 for all *x*. Further, the quorum *k* is defined to be 2,000. Table 2 lists some representative patterns of size two with these parameters. The time sequences are not shown due to the space constraints. These simple patterns can be directly compared with the previous free energy states in the three-dimensional free energy contour maps. These are three-dimensional plots of free energy versus a pair of reaction coordinates or data columns of Table 1.

**Table 2:**
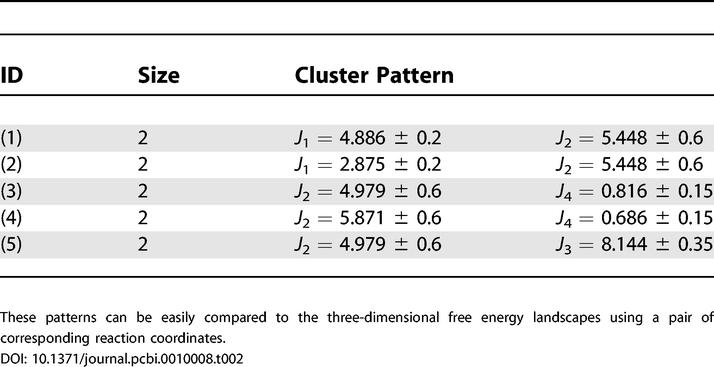
Simple Patterns of Size Two

These patterns can be easily compared to the three-dimensional free energy landscapes using a pair of corresponding reaction coordinates.

One might often want to study detailed patterns or structures in some predefined subregions such as the structures in the unfolded ensemble. More evidence has shown that the protein structures in unfolded states are not fully extended but often have well-defined structures instead [35]. This can also avoid the problem that important patterns in these less populated areas are being overlooked due to a smaller population than the predefined quorum *k*. Thus, some less populated free energy states in free energy landscapes can be recovered by reducing the quorum. Hence, another set of parameters have been used, and here we confine our search to data points with 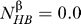 and 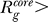 5.0 Å (see Table 1 for definitions of these reaction coordinates) with *k =* 100. Yet another set of parameters have included 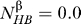 and 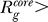 9.0 Å with *k* = 50. A subset of the results is shown later. Thus, this approach might be useful for hierarchical pattern searches that gradually zoom into the predefined subsets of data.

### Analysis of Results

To obtain a representative structure(s) from a set of configurations *c_i_,* the set is partitioned into a minimum number of groups *G_j_* such that for each *G_j_* there exists a representative 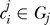 *,* and for each 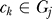 the structure corresponding to *c_k_* is at most 1 Å RMSD from 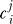 . Thus, each *G_j_* will be represented by a structure corresponding to 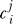 [21,26].

#### Recovering known free energy states.

**Figure 3:**
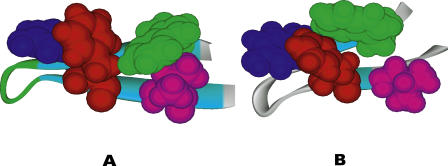
Representative Structures for Two Patterns Hydrophobic residues TRP43, TYR45, PHE52, and VAL54 are represented by spacefill, and the rest of the residues are represented by ribbons. (A) Pattern 1 in Table 2 captures the folded state (F state) in free energy contour map analysis [21]. (B) Pattern 2 in Table 2 captures the partially folded state (P state) in the same free energy contour map.

Obviously, the first question of importance is: Can we recover the previously found free energy states in the new approach? The “time sequence” of each pattern is then used to extract the corresponding conformations of the protein. Figure 3A shows a representative or most populated structure for the first pattern ( 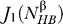 = 4.886 ± 0.2, 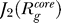 = 5.448 ± 0.6 ) in Table 2. This structure mimics the representative structure from the folded state (F state) in the free energy contour map versus 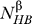 and 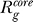 very well. Thus this pattern resembles the F state of the free energy contour map. Similarly, the second pattern of Table 2 ( 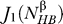 = 2.875 ± 0.2, 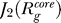 = 5.448 ± 0.6) resembles the partially folded state, P state, in the same free energy landscape. The structures for the two patterns are shown in Figure 3. Thus, our approach recovers the most populated states in the free energy landscape analysis.

The third and fourth patterns in Table 2 also resemble the F state and P state, respectively, in the same free energy contour map versus 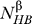 and 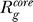 . Numerous other patterns have shown similar results, i.e., recovering various previously found free energy states in the free energy contour maps versus different reaction coordinates. It should be noted, though, that many patterns might be redundant, either because the *δ*() function values given for reaction coordinates are too wide, or because some of the reaction coordinates are highly correlated. For example, the fifth pattern of Table 2 is 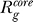 = 4.979 ± 0.6, *R_g =_* 8.144 ± 0.35. Clearly, these two reaction coordinates are highly correlated, since 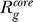 measures the radius of gyration of four key residues out of the total 16 that are measured by *R_g_*. However, for many other cases, it may not be so obvious.

#### Interconnecting various free energy landscapes.

**Figure 4:**
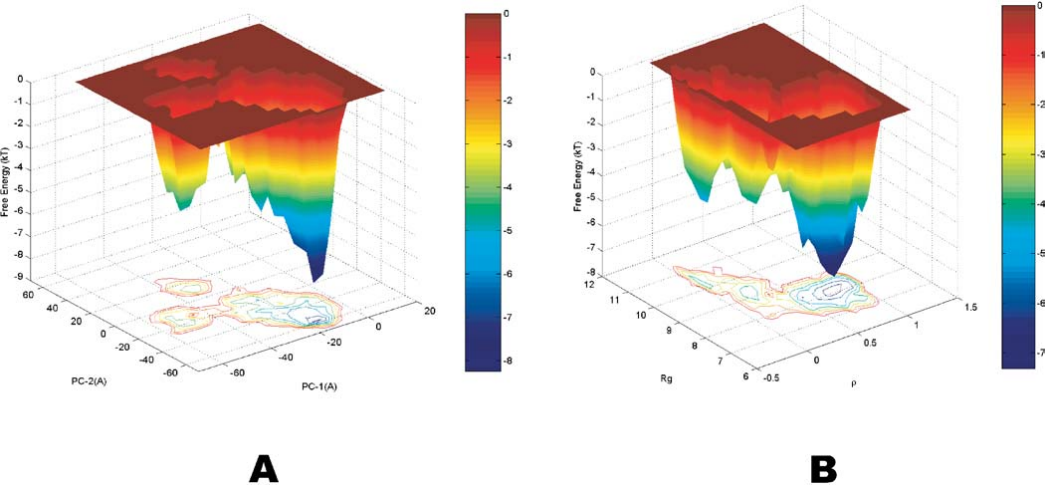
Free Energy Landscapes Free energy landscapes versus (A) the principal components *PC*-1 and *PC*-2, and (B) the fraction of native contact *ρ* and the radius gyration of the peptide *R_g_* at 310 K. The interconnected free energy wells described by the pattern are near −8KT at *PC*-1 = −5.9, PC-2 = −33.6 in (A) and at *ρ* = 0.82, *R_g_* = 8.1 in (B) (see text for more details).

More complicated patterns with many reaction coordinates are also found in the current approach, which had been previously undetected. In the traditional free energy landscape analysis, typically one or two reaction coordinates are used at each time, since a two- or three-dimensional free energy contour map is usually plotted. It is extremely difficult to visualize high-dimensional free energy landscapes in order to identify the free energy basins or barriers. Table 3 lists some of these complicated patterns with up to six reaction coordinates. Of course, as pointed out earlier, some reaction coordinates might be correlated, so the data in each reaction coordinate may not be totally independent. Nevertheless, it still reveals some interesting new findings. First of all, these patterns can interconnect various free energy states in different free energy landscapes. This might not be so obvious in free energy surfaces themselves. For example, the sixth pattern in Table 3, (*R_g_* = 8.144 ± 0.35, *ρ* = 0.815 ± 0.15, *PC*-1 = −5.881 ± 5.0, *PC*-2 = −33.574 ± 16.5, RMSD = 3.292 ± 1.0), interconnects the following two free energy surfaces, one versus *PC*-1and *PC*-2 (Figure 4A), and the other versus *ρ* and *R_g_* (Figure 4B). The states corresponding to the free energy well (of value ≈ −8 KT) near *PC*-1 = −5.9, *PC*-2 = −33.6 in Figure 4A and *ρ* = 0.82, *R_g_* = 8.1 in Figure 4B are the same free energy state since they consist of the same clusters in the same pattern. In this particular case, obviously they all represent the folded state (F state).

**Table 3:**
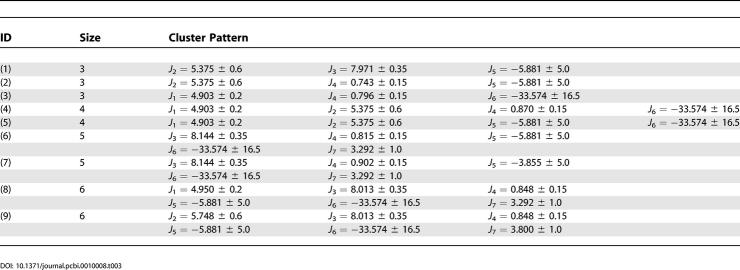
Complex Patterns of Size up to Six

#### Understanding folding mechanism better.

**Figure 5:**
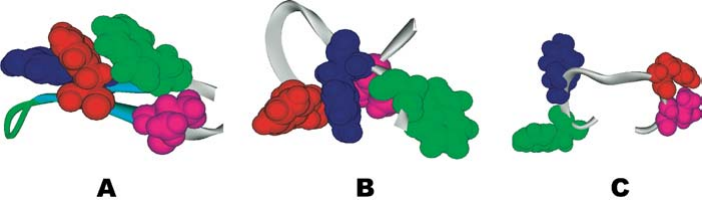
Representative Patterns and Structures (A) Pattern 6 of Table 3, which represents a new class of structures previously overlooked in free energy landscape analysis. (B) Pattern 1 of Table 4, which captures the H state (hydrophobic core formed but no *β*-strand H-bonds) in free energy contour map analysis [21]. (C) Pattern 2 in Table 2 captures the unfolded state (U state) in the same free energy contour map. The hydrophobic residues TRP43, TYR45, PHE52, and VAL54 are represented by spacefill, and the rest are represented by ribbons.

More importantly, the new approach reveals important structures overlooked previously, which might help understand the folding mechanism better. Eaton and coworkers [13,14] proposed a “hydrogen bond zipping” mechanism for this *β*-hairpin, in which folding initiates at the turn and propagates toward the tails by making *β*-strand hydrogen bonds one by one, so that the hydrophobic core, from which most of the stabilization derives, forms relatively late during the folding. In our previous study, we proposed a different folding mechanism, in which this *β*-hairpin undergoes a hydrophobic core collapse first, then makes native *β*-strand hydrogen bonds to make over the free energy loss due to the loss of H-bonds between the backbone atoms and water. Figure 5A shows a representative structure for the eighth pattern in Table 3, ( 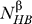 = 4.950 ± 0.2, *R_g_* = 8.013 ± 0.35, *ρ* = 0.848 ± 0.15, *PC*-1 = −5.881 ± 5.0, *PC*-2 = −33.574 ± 16.5, RMSD = 3.292 ± 1.0). The structure shows that all five native *β*-strand H-bonds have been formed, but the hydrophobic core is not completely aligned yet. The loop region also bends toward the hydrophobic core to somewhat offset the non-perfect hydrophobic core. These structures with H-bonds that are formed but with their hydrophobic core not perfectly aligned (RMSDs up to 4 Å) imply that the hairpin can also have a path to form *β*-strand hydrogen bonds before the core is finalized. The current findings indicate that the final hydrophobic core and *β*-strand hydrogen bonds might be formed almost simultaneously. This can also be seen from the low free energy barrier in free energy landscapes as discussed before [21]. Interestingly, Thirumalai et al*.* also found that the lag time between collapse and hydrogen bond formation is very short and the two processes occur nearly simultaneously [32]. It should be pointed out that the turn (loop) formation is critical in this *β*-hairpin folding mechanism, since the hydrophobic core and *β-*strand hydrogen bonds need to be packed or formed at right positions. Interestingly, this is also reported by other groups [15–17]. For example, Gai and coworkers studied a related *β*-hairpin, Trp-zipper hairpin, and found that the rate-limiting event corresponds to the turn formation [15,16]. Moreover, the authors pointed out that a stronger turn-promoting sequence increases the stability of the hairpin primarily by increasing its folding rate, whereas a stronger hydrophobic cluster increases the stability primarily by decreasing its unfolding rate [15,16].

Finally, the patterns of subsets of data in less populated states, such as the unfolded state, are studied in detail by zooming into these regions with a smaller quorum *k* and a different set of *δ*(). As mentioned earlier, more evidence has shown that the protein structures in unfolded states are not fully extended, but often have well-defined structures instead [35]. The first pattern in Table 4 ( 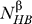 = 0.0, 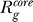 = 5.448 ± 0.5) resembles the previous H-state in free energy contour map versus 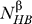 and 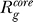 , where the hydrophobic core is largely formed but no native *β*-strand H-bonds have been made yet. Figure 5B shows a representative structure of this pattern, which mimics the structures from previous H-state very well. Figure 5C shows a representative structure for the sixth pattern in Table 4, ( 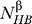 = 0.0, 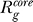 = 9.951 ± 0.35, *ρ* = 0.050 ± 0.15, *PC*-1 = −21.188 ± 15.0, *PC*-2 = 36.517 ± 15.0, RMSD = 9.872 ± 0.8). This is the most populated structure of this *β*-hairpin in unfolded state. Even though not many structural features are found in this structure, it is certainly not fully extended either. Since this is a very small protein with only one secondary structure in the native state, not much has been identified in the unfolded state; for larger and more complicated protein systems, such as lysozymes, more structural features might be expected in the unfolded state as found by recent experiments [35].

**Table 4:**
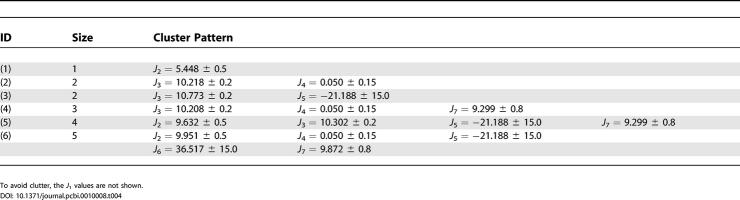
Clusters with (1) *J*_1_ = 0.0, *J*_2_ ≥ 5.0, *k* = 50 and (2) *J*_1_ = 0.0, *J*_2_ ≥ 10.0, *k* = 100

To avoid clutter, the *J*_1_ values are not shown.

### Conclusion

In this paper, we have presented a method to enhance our understanding of protein folding mechanisms. At the heart of this method is a combinatorial pattern-discovery algorithm that analyzes multi-dimensional data from the simulation of the protein folding trajectory. The approach is based on pattern computation, each pattern being defined by a cluster of the reaction coordinates. A small but important protein system, a *β*-hairpin from the C-terminus of protein G, is then used to demonstrate this approach. It is shown that the method not only reproduces the previously found free energy states in free energy contour maps, but also reveals new information overlooked previously in free energy landscape analysis about the intermediate structures and folding mechanism. It is also shown to be useful in making interconnections between various three-dimensional free energy surfaces versus different reaction coordinates and also explains the mechanism behind the folding process. The method also validates the choice of reaction coordinates as the analysis without using free energy values compares well with the ones that use them. The success with *β*-hairpin is very encouraging, and we are currently exploring the application of this method to other larger protein molecules.

As stated in the Introduction section, it is important to study the time correlation between various patterns or states. For example, it is extremely useful to know which pattern or state precedes the other and by how much time. Of course, this requires real-time trajectory data. The current study uses the previous trajectories of REMD, which is a Monte Carlo method; thus, the time sequence in the data points is not real time. After this method's success with the current data, we believe that we will be able to garner time correlation of the patterns, and we are currently investigating this.

## Materials and Methods

We first define the problem at hand and then give a linear time algorithm to solve the problem. The number of clusters can be easily controlled by the use of an appropriate *δ*() function (see below).

### Combinatorial problem description.

In this section, we describe the combinatorial problem. Here, we also make some simple observations that have quite useful and practical implications (such as linear number of *δ*-clusters and so on). They also indicate to the extent different functions (such as the form of *δ*()) can be relaxed without sacrificing the general framework presented in this section. A reader may skip the statements and the proofs of these observations without any loss of continuity. Definitions 1 and 2 identify the pattern discovery or the clustering problem used in this paper, and the Results/Discussion section describes an output-sensitive algorithm to discover them.

First, we begin with a general definition of the *δ*-cluster and *δ*() function and also present the conditions under which the number of patterns are small.

**Definition 1.** (*δ*-cluster, maximal *δ* cluster) Given *δ*() : *R* → *R* ^+^, 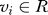 , 1 ≤ *i* ≤ *n* and a quorum *k*. A *δ*-cluster is collection of *i* with 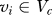 , 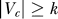 such that if 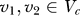 , then 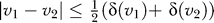 . Further, *V_c_* is maximal if there exists no *V_c_* such that 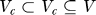 and *V_c_* is a *δ*-cluster.

Although using a general *δ*() function opens the possibility of various pre-processing of the data, it is important to identify a reasonable *δ*() function. We impose the following condition on *δ*(), calling it the **constrained** *δ* **function**. Given any three data elements with *ν*_1 <_ *ν*_2 <_ *ν*_3_, if (*v*_3_ − *δ*(*v*_3_)) ≤ (*v*_1_ + *δ*(*v*_1_)) then (*v*_2_ − *δ*(*v*_2_)) ≥ (*v*_3_ − *δ*(*v*_3_)) and (*v*_2_ + *δ*(*v*_2_)) ≤ (*v*_1_ + *δ*(*v*_1_)).

This is a reasonable condition on an acceptable *δ*() function, as can be seen from the consequence of the imposed constraint in Lemma 1. A multitude of continuous functions satisfy this condition, and in the rest of the paper we will assume that *δ*() function we use also satisfies this condition.

**Lemma 1.** A *δ*-cluster on *ν*_1 <_ *ν*_2 < …_ *ν_n_*

is of the form *ν_i_*_ <_ *ν_i_*_+1_,_…_,*ν_i_*_+*l*_.

Let *V* be a *δ*-cluster with *ν*_min_ (*ν_i_*) as the minimum and *ν*_max_ (*ν_i_*_+*l*_) as the maximum elements. Since *ν*_max_ and *ν*_min_ are in the *δ*-cluster, *ν*_max_ − *δ*(*ν*_max_) ≤ *ν*_min_ + *δ*(*ν*_min_). Thus, for any 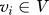 , by the imposed condition, then *ν_i_* − *δ*(*ν_i_*) ≥ *ν*_max_ − *δ*(*ν*_max_) and *ν_i_* − *δ*(*ν_i_*) ≤ *ν*_min_ + *δ*(*ν*_min_):



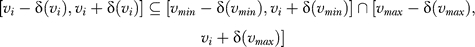



Thus, the containment of the intervals is as shown; hence, for each *ν_i_* , *ν_min_* < *ν_i_ < ν*_max_, 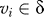 -cluster.

**Lemma 2.** The number of maximal *δ*-clusters is no more than *n* where *δ*() is constrained.

By Lemma 1, any *δ*-cluster is an interval (contiguous elements on the sorted list) on the sorted list of data elements. We will show that any two intervals that correspond to two maximal *δ*-clusters cannot be such that one is contained in the other. Assume the contrary that one is contained in the other. Clearly, by the definition of maximality, the smaller interval is not maximal, leading to a contradiction. As no interval is contained in the other, it is possible to assign a unique element on the sorted data elements to each interval. Thus, the number of intervals cannot exceed the number of data elements, hence the result.

**Corollary 1.** If *δ*(*x*) = *c* for some 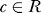 , then the number of *δ*-clusters is no more than *n*.

The bicluster takes into account the different columns or features in the data: the natural definition of such a cluster is given below.

**Definition 2.** (bicluster, maximal bicluster) Given *δ* *_j_*() : *R* → *R* ^+^, quorum *k* and 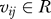 , 1≤ *j≤ m,* 1≤ *i ≤ n*. A bicluster is collection *i* and *j* with 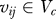 such that for each *j*, 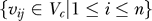 is a *δ_j_*-cluster. Further, *V_c_* is maximal if there exists no additional *i*′ or *j*′ with the corresponding *V_c_* with 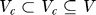 such that *V_c_* is a bicluster.

For ease of reference, the bicluster will be also called a **pattern cluster** since a cluster can be represented by the signature pattern (*J*_1_ = *c_1_*,* J_2_ = c_2_,..., J_L_* = *c_L_*), where 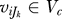 , 1 ≤ *k ≤ L*. These *J*_1_,* J_2_,..., J_L_* represent various reaction coordinates from the protein folding trajectory (shown in Table 1). This representation is more suitable for interpreting the results, as seen in other sections of this paper. The **size** of the bicluster is *L,* and *k* is the number occurrences or **quorum** of the cluster.

**Lemma 3.** The following are a consequence of the maximality constraint: (1) If a collection of *i* is such that 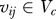 where *V_c_* is a maximal *δ*-cluster for some *j,* then there exist no other maximal *δ*-cluster *V_c_* ≠*V_c_* such that 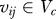 . (2) If a collection of *j* is such that 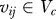 where *V_c_* is a maximal *δ*-cluster for some *j,* then there exist no other maximal *δ*-cluster *V_c_* ≠*V_c_* such that 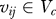 .

**Lemma 4.** Given 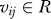 , 1 ≤ *j* ≤ *m,* 1 ≤ *i* ≤ *n*. the number of maximal biclusters is no more than *n* ^2^ *m*.

In a maximal bicluster *V_c_* for some *j,* 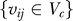 is not necessarily maximal. The number of such clusters by Lemma 2 can be no more than *n^2^*. By Lemma 3, this can belong to only one maximal bicluster. Thus, there can be no more than *n* ^2^ *m* maximal biclusters, since there are *m* columns.

### The linear time algorithm.

Similar descriptions of bicluster detection appear in [36], in which the authors present only an empirical time bound (linear with output size). G. Alexe and P.L. Hammer also present an incremental polynomial time algorithm with a total running time of *O*(*Nnm* ^2^) (personal communication). *N* is the number of patterns in the output, and (*n* × *m*) is the size of the input. In this section, we present an output-sensitive algorithm that computes all the maximal biclusters. The algorithm has two main steps. In the first step, the maximal *δ*-clusters are computed, and in the second step, the maximal biclusters are computed using the clusters of the first step.

**Step 1: Maximal** *δ_j_* **-cluster computation.** For each *j*, 1 ≤ *j* ≤ *m*, compute the maximal *δ_j_* -cluster, 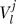 . For simplicity, let the number of these be *L^j^* and the clusters be 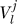 , 1 ≤ *l* ≤ *L^j^* and they are computed as described below. We present a simple algorithm that does a linear scan of the sorted entries *v_ij_* for each fixed *j* using two pointers *i* and *l*: *i* tracks the start of the cluster, and *l* tracks the end of the cluster. The end pointer is incremented until it is no longer a cluster satisfying the *δ*() function, and only then the start pointer is incremented. The pseudocode, *Compute-Cluster(),* describes the maximal *δ*-cluster computations, for each *j*. To avoid clutter, the end-of-input check is not included in the code.

Compute-Cluster()

(1) Sort the *ν_i_*'s to obtain *ν*_1_, *ν*_2_, …, *ν_n_*

(2) *i* ← 1, *l* ← *i* + 1

(3) If 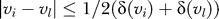


(4) Then *l* ← *l* + 1, go to Step (3)

(5) Else 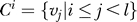 ,

*i* ← *i* + 1, go to Step (3)

Next, for each *ν_ij_*, 1 ≤ *i* ≤ *n*, 1 ≤ *j* ≤ *m,* a set of *δ*-clusters *v*′*_ij_* is computed as follows: 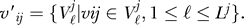


**Step 2: Maximal bicluster computation.** The algorithm in this step is based on the set intersection problem described previously [37] in the context of computing redundant motifs from irredundant ones. The algorithm works on *v*′*_ij_*,1 ≤ *i* ≤ *n*,1 ≤ *j* ≤ *m*, of the last step.

We describe a simple recursive algorithm to solve this problem. This algorithm implicitly constructs a tree in a depth-first manner where (1) each level corresponds to a distinct *j,* hence the height of the tree is *m,* and (2) each non-leaf node at level *l* corresponds to *j* = (m − l) (the root at level 0 corresponds to (*j* = *m*), and has at most (*L^j^ +* 1) children, the ℓth child, 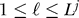 , corresponds to the *δ*-cluster 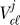 and the very last child ([*L^j^* + 1]th child) ignores the 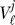 *δ*-clusters. The algorithm is efficient due to the two following factors: (1) use of a data structure (*D* in the pseudocode below) to store the maximal biclusters, so that searching for an arbitrary one can be done quickly, and (2) terminating the tree traversal appropriately. The data structure suggested for use is a tree so that each query takes log *n* time. The terminating condition (line [2.4] of the pseudocode) is such that each leaf node corresponds to either the maximal bicluster defined by the *δ*-clusters (feature values) 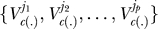 where *j*_1_ ≤ *j*_2_ … ≤ *j_p_* or its variants of the form 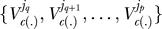 where 1 ≤ *q* ≤ *ρ*.

The pseudocode of the recursive routine *Generate-Set()* shown below, describes the algorithm. Assume a function Add-set (*R,C*), which inserts *R,* a subset of integers between one and *n*, in a tree data structure *D,* along with the accompanying set *C*: then a query of the form if a set *R* exists in *D* takes *O*(log *n*) time. The initial call is *Generate-Set* ({1,2,…,*n*},*φ,m*)).

Generate-Set (*R,C,j)*

(1) If (*j* ≤ 0) then exit

(2) For 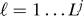


Let 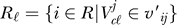


Let 
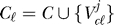


If 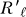 exists in *D* (as (*R*″,*C*″)), add 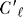 to *C*″

Else

Add-set ( 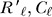 ) to *D*

Generate-Set ( 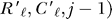


(3) Generate-Set (*R,C,j* − 1)

The maximal biclusters are 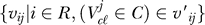 , for each computed (*R,C*) stored in *D*.

### Analysis of the algorithm.

We first show that the algorithm is correct in computing all the maximal biclusters and next show that the algorithm runs in time linear with the size of the output.

**Correctness of the Algorithm.** We first show that each computed (*R,C*) is a bicluster. By the construction, for each *j*, 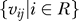 is a *δ*-cluster. Thus (*R,C*) is a bicluster. Next, we have to show that it is maximal. Assume it is not. Then there exists *v_ij_* such that 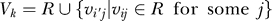 is a bicluster. Hence for each *j,* 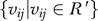 is a *δ*-cluster. Then in the subroutine call *Generate-Set* (*R*,*C*,*i*′) of the pseudocode , this set must have been created, leading to a contradiction. Hence, the assumption is wrong.

Next, assume there exists 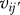 such that 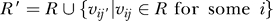 is a bicluster. Hence for each *j*, 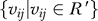 is a *δ*-cluster. Then in Step 3 of the subroutine call *Generate-Set*(*R,C,i*), *V_d_* corresponding to *j*′ must have been included, leading to a contradiction. Hence, the assumption is wrong. Thus, all the computed sets are maximal biclusters. By similar arguments, it is easy to see that if there is any maximal bicluster defined on the data set, it must one of the computed *R*'s.

**Complexity of the Algorithm.** Assume the input elements are *ν_ij_,* 1 ≤ *i* ≤ *n,* 1 ≤ *j* ≤ *m*. Consider the first step of computing the *δ*-clusters for each *j*. The sorting of the elements *ν_i_*, 1 ≤ *I ≤ n* takes *O*(*n* log *n*) time. The algorithm works by scanning the input from left to right, say *i* to *I* + *s,* where the set {*ν_i_* , *ν_i+_*_1_ ,…, *ν_i_*_+*s*_ } is a maximal *δ*-cluster. Then the input is scanned from *i +* 1, *i* + *s +* 1, *i + s +* 2,… onwards and so on. Thus, each data element is visited no more than twice. Assuming the comparison can be made in constant time, this step of the algorithm takes *O*(*n* log *n + n*) =* O*(*n* log *n*) time for each *j*.

Next, consider the second step of computing the maximal biclusters. Notice that the search in Step (2.4) of the subroutine *Generate-Set* can be done in log *n* time. In the recursive-call tree structure (of the subroutine *Generate-Set*), each leaf node corresponds to a maximal bicluster. In a tree, the number of internal nodes is bounded by the number of leaf nodes and each leaf node is hit only as many times as the number of features in each pattern, thus assuming the output size is *N* (the total number of features in all the maximal biclusters) and the second step of the algorithm takes *O*(*N* log *n*) time. Thus, the time taken by the complete algorithm is *O*((*nm* + *N*) log *n*), where *N* is the size of the output and *nm* is the size of the input.

## Abbreviations

PC: principal component
REMD: replica exchange molecular dynamics
RMSD: root mean square deviation
